# Protective effect of silymarin on viability, motility and mitochondrial membrane potential of ram sperm treated with sodium arsenite

**Published:** 2016-06

**Authors:** Farzaneh Eskandari, Hamid Reza Momeni

**Affiliations:** *Department of Biology, Faculty of Science, Arak University, Arak, Iran.*

**Keywords:** *Spermatozoa*, *Arsenic*, *Silymarin*

## Abstract

**Background:**

Sodium arsenite can impair male reproductive function by inducing oxidative stress. Silymarin is known as a potent antioxidant.

**Objective:**

This study was performed to investigate if silymarin can prevent the adverse effect of sodium arsenite on ram sperm viability, motility and mitochondrial membrane potential.

**Materials and Methods:**

Epidydimal spermatozoa obtained from ram were divided into five groups: 1) Spermatozoa at 0 hr, 2) spermatozoa at 180 min (control), 3) spermatozoa treated with sodium arsenite (10 μM) for 180 min, 4) spermatozoa treated with silymarin (20 μM) + sodium arsenite (10 μM) for 180 min and 5) spermatozoa treated with silymarin (20 μM) for 180 min. MTT assay and Rhodamine 123 staining were used to assess sperm viability and mitochondrial membrane potential respectively. Sperm motility was performed according to World Health Organization (WHO) guidelines.

**Results:**

Viability (p<0.01), nonprogressive motility (p<0.001) and intact mitochondrial membrane potential (p<0.001) of the spermatozoa were significantly decreased in sodium arsenite treated group compared to control group. In silymarin + sodium arsenite group, silymarin could significantly reverse the adverse effect of sodium arsenite on these sperm parameters compared to sodium arsenite group (p<0.001). In addition, the application of silymarin alone for 180 minutes could significantly increase progressively motile sperm (p<0.001) and decrease non motile sperm (p<0.01) compared to the control.

**Conclusion:**

Silymarin could compensate the adverse effect of sodium arsenite on viability, nonprogressive motility and mitochondrial membrane potential of ram sperm.

## Introduction

Nowadays infertility has become one of the critical problems for couples. Arsenic as a toxic metal and environmental contaminant could be a risk factor for male fertility. The use of arsenic-containing herbicides, insecticides, rodenticides, and preservatives and by-products of fossil fuels is enough to endanger men fertility (1). 

Arsenic can induce male reproductive toxicity through damage in testes structure , sex hormones imbalance and decrease in testes and accessory sex organ weights as well as reduction in epididymal sperm count, normal morphology, viability and motility (2-6). Arsenic is proposed to exert its cytotoxicity by free radicals generation and the activation of oxidative sensitive signaling pathways (7, 8). Therefore, the use of natural antioxidants could be a possible strategy for reducing oxidative stress in body. Silymarin is extracted from milk thistle *(Silybum marianum*) seeds (9). This compound is a polyphenolic flavonoid with a potent antioxidant property which not only acts as free radical scavenger but also increases the capacity of cell antioxidant enzymes (10-12). This plant antioxidant might therefore be considered as an alternative to ameliorate the toxic effect of arsenite and infertility mediated by this pollutant.

Viability and motility are considered as the most important parameters of mature sperm which indicate its structural and functional quality to move toward an egg for successful fertilization. Thus a focus on mitochondria integrity, which reflects the metabolic status of sperm to provide energy for survival and movement, can give information about the health of sperm for fertilization process. No doubt, factors that impair mitochondrial integrity and energy production of sperm endanger its health to reach the egg. 

Due to the toxic effect of arsenic on reproduction and fertility, this study was carried out to investigate if silymarin as a potent antioxidant can prevent the adverse effect of sodium arsenite on viability, motility and mitochondrial membrane potential of ram sperm.

## Materials and methods

**Epididimal sperm collection and** **treatments**

In this experimental study, Farahani's ram testes were received from Arak slaughterhouse immediately after ram daily slaughter for public consumption and transferred to the research laboratory under standard conditions. The ethical issues in the use of animals in research were observed. The experiment were approved by the ethical committee at Arak University A few incisions were made in the caudal epididymis and spermatozoa were then washed into a sterile Falcon tube by Ham's F10 medium (Sigma, USA). Firstly sperm number and sperm motility were determined, according to World Health Organization protocol (WHO), to estimate sperm quality (13).

High quality sperm samples were then used for experiments. The sperm samples were separated in eppendorf tubes as each tube contained 5×10^6^ spermatozoa and divided into five groups (n=6): 1. Spermatozoa at 0 hr, 2. Control spermatozoa, 3. Spermatozoa treated with sodium arsenite (10 μM, Merck, Germany), 4. Spermatozoa treated with silymarin (20 μM, Sigma, USA) + sodium arsenite (10 μM) and 5. Spermatozoa treated with silymarin (20 μM). Samples 2-5 were kept at 37^o^C in a CO_2_ incubator for 180 min.

**Sperm viability**

The MTT, 3-(4, 5-dimethylthiazol-2-yl)-2, 5-diphenyl tetrazolium bromide, assay was used to assess viability. This assay was performed based on a method described by Mosmann (14). In brief, 10 µl of MTT (Sigma, USA) stock solution (5 mg/ml ham's F10) was added to each tube containing sperm suspension and incubated at 37^o^C in CO_2_ incubator for 1 hr. The tubes centrifuged at 6000 rpm for 6 min and the precipitate was dissolved in 200 µl dimethyl sulfoxide (DMSO). The solution was then centrifuged at 4000 rpm for 4 min. 100 µl of the purple solution were transferred into a 96-well plate and absorbance was measured using ELISA reader (SCO diagnostic, Germany) at 505 nm. Optical density of sample was then used for calculating sperm viability percentage .

**Sperm motility**

Evaluation of sperm motility was done according to WHO guidelines (13). In brief, 10 µl of sperm suspension was placed on semen analysis chamber. Minimum of five microscopic fields was evaluated to estimate sperm motility on at least 200 spermatozoa for each sample. The percentage of sperm motility was evaluated for following motion patterns: progressive motile sperm (PMS), nonprogressive motile sperm (NPMS) and nonmotile sperm (NMS).

**Sperm mitochondrial membrane potential**

Mitochondrial membrane potential (MMP) was measured using Rhodamine 123 which is a cationic, cell permeable and fluorescent dye. It is rapidly absorbed by active mitochondria and has no toxic effect (15). For staining, 5 µl of Rhodamine 123 (Sigma, USA) stock solution (1 mg/ml) was added to each tube containing sperm suspension and kept at 25^o^C for 10 min in dark place. Tubes were centrifuged at 300 gr for 10 min and 1 ml Phosphate Buffered Saline was then added to remainder precipitate. Thin smears were then prepared and observed under an Olympus fluorescence microscope at 1000× magnification (16). At least 100 spermatozoa were counted in at least 5 different microscopic fields per slide and expressed as percentage. 

**Statistical analysis**

The results expressed as mean±SD for six samples per group. One-Way analysis of variance (ANOVA) followed by Tukey's test was used to assess data statistical significance. p<0.05 was considered significant.

## Results

**Sperm viability**

**Figure1:**
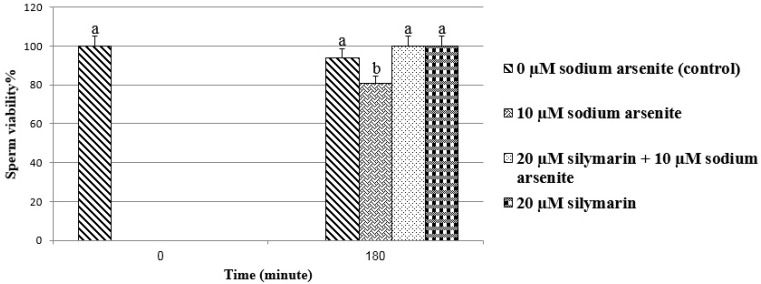
Evaluation of ram sperm viability by MTT assay. Means with the same words do not differ significantly. Mean ± SD, one way ANOVA, Turkey’s test, n=6 per group, (p<0.05

The percentage of sperm viability in sodium arsenite group was significantly decreased in comparision with control group (spermatozoa at 180 min) (p<0.01). In silymarin+ sodium arsenite group, silymarin could significantly (p<0.001) reverse the adverse effect of sodium arsenite on sperm viability compared to sodium arsenite group (Figure 1).

**Sperm motility**

**Figure 2:**
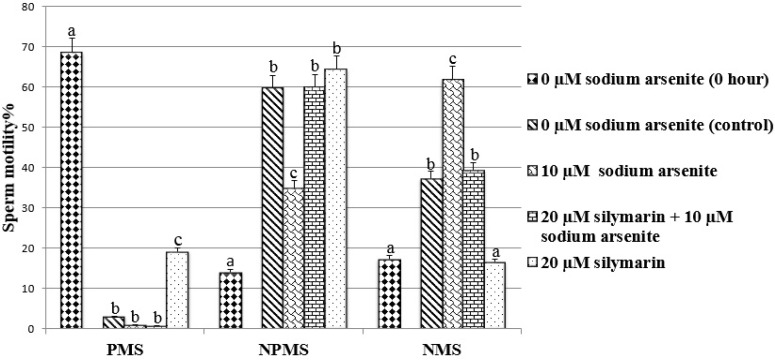
Evaluation of ram sperm motility. PMS: progressive motile sperm, NPMS: nonprogressive motile sperm and NMS: nonmotile sperm. Means with the same words do not differ significantly. Mean±SD, one way ANOVA, Turkey’s test, n=6 per group, (p<0.05

No significant differences were found in PMS percentage in the group treated with sodium arsenite compared to the control, while the percentage of NPMS was significantly decreased (p<0.001) and the percentage of NMS was significantly increased (p<0.001) compared to the control (Figure 2). Spermatozoa treated with silymarin + sodium arsenite showed no change in PMS percentage compared to sodium arsenite group. However, silymarin could significantly reverse the adverse effect of sodium arsenite on the NPMS and NMS percentage (p<0.001) (Figure 2). 

In addition, in silymarin group, PMS percentage was significantly increased (p<0.001) while the NMS percentage was decreased significantly (p<0.001) compared to the control (Figure 2).

**Sperm mitochondrial membrane potential**

**Figure 3:**
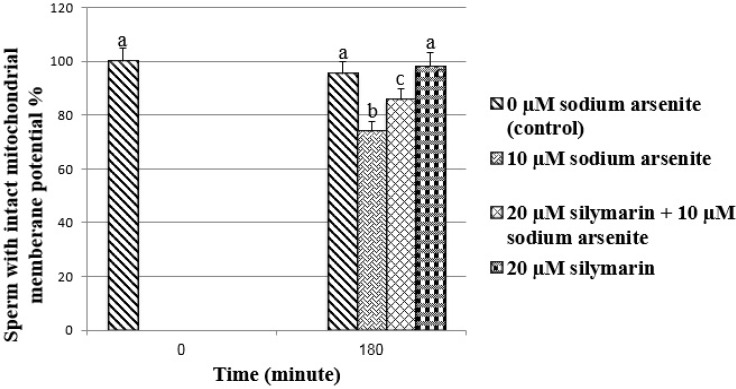
Evaluation of ram sperm mitochondrial membrane potential by rhodamine 123 staining. Means with the same words do not differ significantly. Mean±SD, one way ANOVA, Turkey’s test, n=6 per group (p<0.05

**Figure 4:**
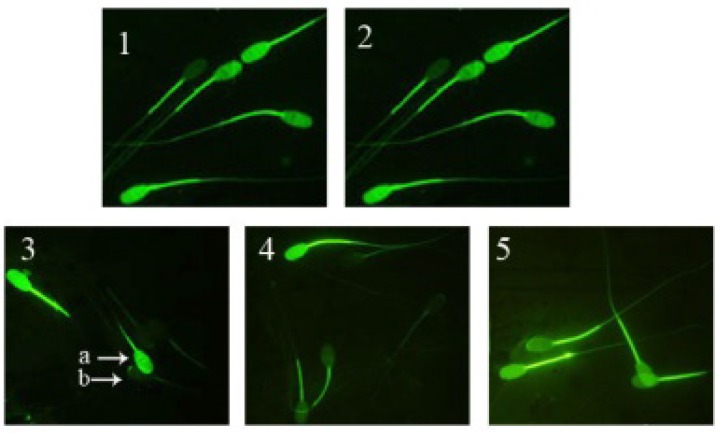
Ram sperm mitochondrial membrane potential (MMP), stained with rhodamine 123 staining. 1. Spermatozoa at 0 hour, 2. Spermatozoa at 180 minutes (control), 3. Spermatozoa treated with sodium arsenite (10 μM) for 180 minutes, 4. Spermatozoa treated with silymarin (20 μM) + sodium arsenite (10 μM) for 180 minutes and 5. Spermatozoa treated with silymarin (20 μM) for 180 minutes. Magnification: 1000×. a) Sperm with intact MMP shows brilliant green. b) Sperm with damaged MMP remained colorless

The application of sodium arsenite, significantly decreased (p<0.001) the sperm percentage of with intact MMP compared to the control. In silymarin group+ sodium arsenite, silymarin could significantly (p<0.001) ameliorate the toxic effect of sodium arsenite on MMP compared to sodium arsenite group (Figure 3, 4).

## Discussion

In this study the adverse effect of sodium arsenite on viability, motility and mitochondrial membrane potential of ram epididymal sperm was examined. Also the protective effect of silymarin on these parameters in sodium arsenite treated spermatozoa was tested. Evaluation of sperm mitochondrial integrity and metabolic status in the health and pathology could provide information about sperm health as well as sperm motility and viability. Although visual estimation of viable sperm using the traditional methods such as eosin-nigrosin and trypan blue staining are simple and inexpensive, the results can be influenced by experience of analyst (17). 

In addition, these methods just assess membrane integrity and provide no information about sperm mitochondrial capacity and metabolic status. MTT reduction assay which evaluates viability in wide variety of cells could be used for the assessment of sperm mitochondrial capacity to estimate sperm fertility (14, 18-20). In this context, MTT assay has been used to evaluate sperm viability in stallion, bull and boar (21-23). In the present study, MTT method was used, for the first time, to quantitatively assess ram sperm viability. In this method, the yellow water-soluble tetrazolium dye, MTT, is reduced by the active mitochondria dehydrogenases to an insoluble purple formazan. Thus, the amount of formed formazan can be determined spectrophotometrically and serves as an number estimation of active mitochondria and hence the number of living cells (24).

Our results showed a significant decrease in viability percentage (p<0.01) and intact MMP (p<0.001) in sodium arsenite treated spermatozoa. In addition, this toxicant was negatively affected the percentage of NPMS and NMS (p<0.001). This result was in agreement with previous findings on sperm viability and motility of rat as well as mice sperm motility under in-vivo conditions. Mitochondria might be the most important target for arsenic toxicity (5, 25). Arsenic impairs electron transfer chain and subsequently induces the formation of reactive oxygen species (ROS) which in turn exert lipid peroxidation (26). 

Arsenic has also an ability to complex with protein's SH groups, thus depleting the mitochondrial glutathione (GSH) level which is not only an antioxidant but also is an essential factor for maintenance of mitochondrial proteins thiol groups in the reduced state (27, 28). Under oxidative stress, oxidation of thiol groups caused by arsenic induces a dysfunction in mitochondrial permeability transition pore (29). The opening of the pores causes the movement of unlimited proton into the mitochondria followed by collapse of MMP, leads to uncoupling of oxidative phosphorylation and further reduction of ATP production (30).

On the other hand, a change in mitochondria permeability results in the release of mitochondrial cytochrome C, leading to the activation of caspase-9 and caspase-3 in the mitochondrial pathway (31). We therefore hypothesized that toxic effect of sodium arsenite on the sperm viability, motility and intact MMP could be due to the ability of this toxicant in oxidative stress induction . If our hypothesis was true, the application of antioxidant should reverse hazardous effect of sodium arsenite on these sperm parameters. Interestingly, we showed that in spermatozoa treated with silymarin + sodium arsenite, silymarin as a potent antioxidant, could significantly compensate the adverse effect of sodium arsenite on the percentage of viability (p<0.001), nonprogressive motility (p<0.001) and intact MMP (p<0.001) of ram sperm compared to sodium arsenite group (10). 

Because of high concentration of polyunsaturated fatty acids and low antioxidant enzymes, mammalian spermatozoa are susceptible to lipid peroxidation mediated by oxidative stress (32). Therefore, it could be speculated that silymarin by improving the activity of sperm antioxidant defense system exerted its antioxidant role on the sodium arsenite mediated toxicity. The assessment of antioxidant defense system enzymes in the mentioned groups is suggested to provide insights toward this possible mechanism.

The incubation of spermatozoa for 180 min (control group) caused a significant decrease in PMS percentage as well as significant increase in NPMS and NMS percentage compared to spermatozoa at 0 hr. The prolonged in-vitro incubation of human spermatozoa was shown to induce a time dependent loss of motility and some parameters related to sperm motion (33). Such decrease has been suggested to be associated with ROS generation (34). 

It is therefore possible that the motion patterns deterioration of ram sperm during 180 min incubation, under aerobic conditions, might be attributed by oxidative attack or an unbalanced between sperm oxidation and antioxidant defense system. To support this idea, we showed that the application of silymarin alone for 180 min significantly increased the PMS percentage (p<0.001) and decreased the NMS percentage (p<0.001) compared to the control. This effective result of silymarin might also be due to its antioxidant role in improving the capacity of sperm antioxidant defense system.

## Conclusion

Our results indicate that sodium arsenite has a negative influence on sperm viability, motility and intact MMP. In addition, silymarin is able to compensate the adverse effects of sodium arsenite on these parameters.

## Note

***This article extracted from M.Sc. Thesis. (Farzaneh Eskandari)***

## References

[1] Flora SJS, Dube SN, Arora U, Kannan GM, Shukla MK, Malhotra PR (1995). Therapeutic potential of meso 2, 3-dimercaptosuccinic acid or 2, 3-dimercaptopropane 1-sulfonate in chronic arsenic intoxication in rats. Biometals.

[2] Soleimani Mehranjani S, Hemadi M (2007). The effects of sodium arsenite on the testis structure and sex hormones in vasectomised rats. Iran J Reprod Med.

[3] Ahmad I, Hussain T, Akthar K (2008). Arsenic induced microscopic changes in rat testis. Prof Med J.

[4] Jana K, Jana S, Samanta PK (2006). Effects of chronic exposure to sodium arsenite on hypothalamo-pituitary-testicular activities in adult rats: possible an estrogenic mode of action. Reprod Biol Endocrinol.

[5] Pant N, Murthy RC, Srivastava SP (2004). Male reproductive toxicity of sodium arsenite in mice. Hum Exp Toxicol.

[6] Mukherjee S, Mukhopadhyay P, others (2009). Studies on arsenic toxicity in male rat gonads and its protection by high dietary protein supplementation. Al Ameen J Med Sci.

[7] Lee T-C, Ho I-C (1994). Differential cytotoxic effects of arsenic on human and animal cells. Environ Health Perspect.

[8] Valko M, Morris H, Cronin MTD (2005). Metals, toxicity and oxidative stress. Curr Med Chem.

[9] Khan SA, Ahmed B, Alam T (2006). Synthesis and antihepatotoxic activity of some new chalcones containing 1, 4-dioxane ring system. Pak J Pharm Sci.

[10] Kohno H, Tanaka T, Kawabata K, Hirose Y, Sugie S, Tsuda H (2002). Silymarin, a naturally occurring polyphenolic antioxidant flavonoid, inhibits azoxymethane-induced colon carcinogenesis in male F344 rats. Int J Cancer.

[11] Kiruthiga P V, Shafreen RB, Pandian SK, Arun S, Govindu S, Devi KP (2007). Protective effect of silymarin on erythrocyte haemolysate against benzo (a) pyrene and exogenous reactive oxygen species (H2O2) induced oxidative stress. Chemosphere.

[12] Soto C, Recoba R, Barrón H, Alvarez C, Favari L (2003). Silymarin increases antioxidant enzymes in alloxan-induced diabetes in rat pancreas. Comp Biochem Physiol Part C Toxicol Pharmacol.

[13] Organization WH (1999). WHO laboratory manual for the examination of human semen and sperm-cervical mucus interaction.

[14] Mosmann T (1983). Rapid colorimetric assay for cellular growth and survival: application to proliferation and cytotoxicity assays. J Immunol Methods.

[15] Scaduto Jr RC, Grotyohann LW (1999). Measurement of mitochondrial membrane potential using fluorescent rhodamine derivatives. Biophys J.

[16] De Vantéry Arrighi C, Lucas H, Chardonnens D, De Agostini A, others (2009). Removal of spermatozoa with externalized phosphatidylserine from sperm preparation in human assisted medical procreation: effects on viability, motility and mitochondrial membrane potential. Reprod Biol Endocrinol.

[17] Klimowicz-Bodys MD, Batkowski F, Ochrem AS, Savič MA (2012). Comparison of assessment of pigeon sperm viability by contrast-phase microscope (eosin-nigrosin staining) and flow cytometry (SYBR-14/propidium iodide (PI) staining) [evaluation of pigeon sperm viability]. Theriogenology.

[18] Campling BG, Pym J, Galbraith PR, Cole SPC (1988). Use of the MTT assay for rapid determination of chemosensitivity of human leukemic blast cells. Leuk Res.

[19] Carmichael J, DeGraff WG, Gazdar AF, Minna JD, Mitchell JB (1987). Evaluation of a tetrazolium-based semiautomated colorimetric assay: assessment of radiosensitivity. Cancer Res.

[20] Levitz SM, Diamond RD (1985). A rapid colorimetric assay of fungal viability with the tetrazolium salt MTT. J Infect Dis.

[21] Aziz DM, Ahlswede L, Enbergs H (2005). Application of MTT reduction assay to evaluate equine sperm viability. Theriogenology.

[22] Aziz DM (2006). Assessment of bovine sperm viability by MTT reduction assay. Anim Reprod Sci.

[23] Park CS, Kim MY, Yi YJ, Chang YJ, Lee SH, Lee JJ (2004). Liquid boar sperm quality during storage and in vitro fertilization and culture of pig oocytes. Asian Australas J Anim Sci.

[24] Byun JW, Choo SH, Kim HH, Kim YJ, Hwang YJ, Kim DY (2008). Evaluation of boar sperm viability by mtt reduction assay in beltsville thawing solution extender. Asian Australas J Anim Sci.

[25] Momeni HR, Eskandari N (2012). Effect of vitamin E on sperm parameters and DNA integrity in sodium arsenite-treated rats. Iran J Reprod Med.

[26] Hosseini M-J, Shaki F, Ghazi-Khansari M, Pourahmad J (2013). Toxicity of arsenic (III) on isolated liver mitochondria: A new mechanistic approach. Iran J Pharm Res.

[27] Lash LH (2006). Mitochondrial glutathione transport: physiological, pathological and toxicological implications. Chem Biol Interact.

[28] Zhang F, Xu Z, Gao J, Xu B, Deng Y (2008). In vitro effect of manganese chloride exposure on energy metabolism and oxidative damage of mitochondria isolated from rat brain. Environ Toxicol Pharmacol.

[29] Pourahmad J, Hosseini M-J, Eskandari MR, Shekarabi SM, Daraei B (2010). Mitochondrial/lysosomal toxic cross-talk plays a key role in cisplatin nephrotoxicity. Xenobiotica.

[30] Shen ZY, Shen J, Cai WJ, Hong C, Zheng MH (2000). The alteration of mitochondria is an early event of arsenic trioxide induced apoptosis in esophageal carcinoma cells. Int J Mol Med.

[31] Green DR, Reed JC (1998). Mitochondria and apoptosis. Sci Pap Ed.

[32] Vernet P, Aitken RJ, Drevet JR (2004). Antioxidant strategies in the epididymis. Mol Cell Endocrinol.

[33] Chomsrimek N, Choktanasiri W, Wongkularb A, O-Prasertsawat P (2008). Effect of time between ejaculation and analysis on sperm motility. Thai Obs Gynaecol.

[34] Calamera JC, Fernandez PJ, Buffone MG, Acosta AA, Doncel GF (2001). Effects of long-term in vitro incubation of human spermatozoa: functional parameters and catalase effect. Andrologia.

